# Coral cell separation and isolation by fluorescence-activated cell sorting (FACS)

**DOI:** 10.1186/s12860-017-0146-8

**Published:** 2017-08-29

**Authors:** Benyamin Rosental, Zhanna Kozhekbaeva, Nathaniel Fernhoff, Jonathan M. Tsai, Nikki Traylor-Knowles

**Affiliations:** 1 Institute for Stem Cell Biology and Regenerative Medicine, Stanford University School of Medicine, Stanford, CA 94305 USA; 2 Department of Pathology, Hopkins Marine Station, Stanford University, 120 Ocean View Blvd, Pacific Grove, CA 93950 USA; 3 University of Miami, Rosenstiel School of Marine and Atmospheric Science, 4600 Rickenbacker Causeway, Florida, 33149 USA

**Keywords:** FACS, Coral, Cell isolation, Cell sorting, Cellular labeling

## Abstract

**Background:**

Generalized methods for understanding the cell biology of non-model species are quite rare, yet very much needed. In order to address this issue, we have modified a technique traditionally used in the biomedical field for ecological and evolutionary research. Fluorescent activated cell sorting (FACS) is often used for sorting and identifying cell populations. In this study, we developed a method to identify and isolate different cell populations in corals and other cnidarians.

**Methods:**

Using fluorescence-activated cell sorting (FACS), coral cell suspension were sorted into different cellular populations using fluorescent cell markers that are non-species specific. Over 30 different cell markers were tested. Additionally, cell suspension from *Aiptasia pallida* was also tested, and a phagocytosis test was done as a downstream functional assay.

**Results:**

We found that 24 of the screened markers positively labeled coral cells and 16 differentiated cell sub-populations. We identified 12 different cellular sub-populations using three markers, and found that each sub-population is primarily homogeneous. Lastly, we verified this technique in a sea anemone, *Aiptasia pallida*, and found that with minor modifications, a similar gating strategy can be successfully applied. Additionally, within *A. pallida*, we show elevated phagocytosis of sorted cells based on an immune associated marker.

**Conclusions:**

In this study, we successfully adapted FACS for isolating coral cell populations and conclude that this technique is translatable for future use in other species. This technique has the potential to be used for different types of studies on the cellular stress response and other immunological studies.

**Electronic supplementary material:**

The online version of this article (10.1186/s12860-017-0146-8) contains supplementary material, which is available to authorized users.

## Background

### Coral reefs to coral cells

Reef building corals are extremely important organisms for marine ecosystems. Corals are diploblastic cnidarians that live in shallow, warm tropical regions, and are critical contributors to reef ecosystems that host much of the ocean’s biodiversity [1, 2]. They provide billions of dollars in revenue for coastal and island countries; however, despite this great importance they are under tremendous threat from anthropogenic climate change [3, 4]. Previous estimates have found that 32.8% of corals assigned to International Union for Conservation of Nature (IUCN) are in danger of extinction [4]. Given their economical and environmental importance, it is imperative to gain a better understanding of how corals react to climate change.

It is well understood that corals will be under great stress under the projected climate change conditions [5]. Reef building corals have an obligate symbiosis with a symbiotic dinoflagellate, *Symbiodinium,* and this relationship is one of the most well characterized cellular interactions in coral cell biology*.* When stressed this relationship breaks down and disrupts the intracellular relationship of *Symbiodinium* and its coral host. This process is called bleaching and it further contributes to the coral host’s stress.

Previously, many coral cellular studies have focused on coral host uptake of the *Symbiodinium* [6, 7], the breakdown of the coral host-*Symbiodinium* relationship [8–16], cellular calcification mechanisms [17–25], cell culture techniques [26–29], and the identification of the intracellular pH relationship between coral host cells and *Symbiodinium* [30–32]. Additionally, flow cytometry has been used to quantify *Symbiodinium* cells, and assay for apoptosis [33, 34]. Lastly, many cellular studies on corals have focused on the histological aspects of stress response and disease of the whole organism [35, 36].

However, other than the breakdown of the relationship between *Symbiodinium* and coral host cell during heat induced stress, little is understood about the role of other cell types during the cellular stress response. Previous studies have found that other cell types including cnidocytes, a family of stinging cell types found only in cnidarians, and other gastrodermal cells may be critical for the heat induced stress response in corals [9]. Additionally, there is little information on the presence of immune-like cells called amoebocytes, in the scleractinian (stony or hard) corals. Previous characterization of amoebocytes was done in the gorgonian coral, *Swiftia exserta,* a non-scleractinian coral [37], and in scleractinian corals, amoebocytes have been identified by histology [38]. In order to address these gaps, we have developed a protocol that uses fluorescence-activated cell sorting (FACS) to efficiently sort cells into different populations based on natural fluorescence and fluorescent cell dyes, allowing us to collect them for further analysis.

### Coral cell types

Corals have two tissue layers, an outer epidermis and internal gastrodermis. These tissue layers are separated by a mesoglea, which harbors multiple cell types including secretory, amoeboid, and reproductive cells [39]. Many cell types reside within the epidermis including ciliated column, secretory, sensory motor neuron, interneuron, neurosecretory, sensory cells, cnidocytes, and flagellated columnar cells [39]. The cell types in the gastrodermis include cuboidal, absorptive, secretory, squamous, columnar, anchoring, flagellated columnar, flagellated cuboidal, spindle shaped, sensory cells, motor neurons, interneurons, neurosecretory and *Symbiodinium* (algal cells which live within the coral gastrodermal cells) [39]. In addition to the endosymbiotic algal cells, there is also some evidence for endosymbiotic bacteria that live within coral tissue layers, however little is understood about their role and function in the coral [40].

### Fluorescence-activated cell sorting (FACS)

Flow cytometry is a powerful technique used to distinguish and characterize cell types, including live cells. This technique, which has been used primarily in biomedical and immunological research, utilizes lasers to analyze and sort different cell types in real time based on specific properties of ﻿the cell. Applications of FACS include clinical analysis, cell purification, functional assays, and pathogen detection [41–47]. Although these techniques have not been widely applied to many non-medical systems, they are a powerful methods for cell type discovery and cell activity in comparative and evolutionary research. Furthermore, isolation of different cells, based on general properties (e.g. lectins, enzymes, size and granularity) that are not antibody- based can successfully be used in separating different cell populations in non-model species and these distinct cell populations are different functionally and physiologically [48–51].

Here, we have developed a method to separate coral cell populations by utilizing cell markers that are non- species specific. This powerful technique allows for cellular differentiation in real time. Using this technique a number of cellular functions can be measured including free radical production, immune properties, intracellular enzymatic activity, and compound uptake. This technique can also be used to separate specific cell populations for gene expression studies, which will allow for more targeted studies of the coral stress response. In this report, we present the methods of cell sorting, and strategies for distinguishing specific coral cells, and specific cell markers for characterizing coral cell types. Additionally, we tested this technique on a symbiotic anemone, *Aiptasia pallida,* as confirmation that this sorting strategy can be applied to other species. Lastly, we tested phagocytosis, as a downstream functional assay on sorted cells expressing high levels of lysolitic vesicles.

## Methods

### Coral fragment collection and coral cell dissociation

Fragments of *Pocillopora damicornis* were obtained from the Monterey Bay Aquarium (MBA) in partnership with the Tropical Coral Propagation program. These corals, which had previously been obtained from illegal shipments, were confiscated by the U.S. Fish and Wildlife Service and donated to MBA for propagation and research.

To prepare the coral cell suspension for FACS analysis, *P. damicornis* fragments of 1–4 cm in length were scraped off of the soft tissue with fine blade into a petri dish containing staining media: 3.3× PBS (without Ca and Mg), 2% FCS (vol/vol) and 20 mM Hepes, pH 7.4. The soft tissue in the petri dish was dissociated with a fine blade into cell suspensions and filtered through a 40 μm mesh. Using the plunger of a sterile 1 ml syringe, cell suspensions were additionally dissociated on the mesh, and washed and collected in staining media. The entire process took approximately 10 min per fragment and was done on ice to minimize damage to the cells and lower their metabolism. Cells were then washed by centrifugation at 500 x g at 4 °C for 5 min. Cells were counted using a hemocytometer and trypan blue diluted 1:10 (ThermoFisher Sientific), a cell viability dye that excludes dead cells. The trypan blue was diluted 1:10 (compared to mammalian cells usage) to 0.04%, due to aggregate formation at high salinity. 

These same methods were then tested in a strain of *A. pallida* containing *Symbiodinium* and a aposymbiotic *A. pallida* strain (strain that is raised without *Symbiodinium*). These strains were kindly provided by the J. R. Pringle mariculture facility at Stanford University [52].

### Cell labeling, phagocytosis and FACS analysis

Cell labeling was done in 96 well U-shaped plates with 10^5^ cells/well in 50 μl of staining media for 30 min at 20 °C. The concentration of the different labeling material is indicated in Table 1. The suspension was then washed once with staining media and re-suspended in 200 μl staining media containing propidium iodide (5 μg/ml) or DAPI (3 μM) prior to FACS reading. For determining the gating of cells and debris, sorting and observation by light microscopy was done several times, and also gating of other invertebrate species was used for validation [48]. The smallest cell size that was in the gate was approximately 4 μm.

**Table 1:** Screened compounds to differentiate coral cells

Compound Name	Company and Catalog	Fluorescent Indicator	Label	Differ. Cells (*Symb.*+)	Conc.
3-dodecanoyl-NBD Cholesterol	CC: 13220	Cholesterol uptake	N	N (N)	1 μM
MeOSuc-Ala-Ala-Pro-Val-AMC	CC: 14907	Elastase activity	N	N (N)	1 μM
Dihydroethidium	CC: 12013	Intracellular superoxide	Y	Y (Y)	1 μM
Pyronin Y	CC: 14488	dsRNA	Y	Y (Y)	0.2 μM
ThioFluor623	CC: 13083	Intracellular thiols	Y	Y (Y)	1 μM
Rhodamine Phenylglyoxal	CC: 16172	Protein citrullination	Y	Y (Y)	0.2 μM
CAY10455	CC: 10005072	Arachidonoyl ethanolamide uptake	Y	Y (N)	0.2 μM
Dibenzylfluorescein	CC: 16808	Cytochrome P450 activity	Y	N (Y)	0.2 μM
BCECF-Acetoxymethyl ester	CC: 15922	Intracellular pH	Y	Y (Y)	0.2 μM
NBD-FTY720 phenoxy HCl	CC: 16841	Sphingosine-1-phosphate	Y	N (N)	1 μM
10-Acetyl-3,7-dihydroxyphenoxazine	CC: 10010469	Hydrogen Peroxide/Peroxidase	Y	N (N)	1 μM
2-NBDG	CC: 11046	Glucose uptake	Y	N (N)	1 μM
1-NBD-decanoyl-2-decanoyl-sn-Glycerol	CC: 9000341	Diacylglycerol uptake	Y	N (N)	1 μM
(Z-Ala-Ala-Ala-Ala)2Rh110	CC: 11675	Elastase activity	N	N (N)	1 μM
5-Cyano-2,3-di-(p-tolyl)tetrazolium Cl	CC: 15926	Aerobic metabolic activity	N	N (N)	1 μM
Coumarin Boronic Acid pinacolate ester	CC: 10818	Peroxynitrite	N	N (N)	1 μM
Garcinol	CC: 10566	Histone acetyltransferase inhibitor	N	N (N)	1 μM
WSP-1	CC: 11179	Hydrogen Sulfide	N	N (N)	1 μM
Zinpyr-1	CC: 15122	Intracellular Zinc ion	Y	N (Y)	0.2 μM
DPPP	CC: 62237	Hydroperoxides	N	N (N)	1 μM
Acridine Orange	CC: 14338	DNA dye	Y	Y (Y)	0.2 μM
Fura-PE3-Acetoxymethyl ester	CC: 15366	Intracellular Calcium	Y	Y (N)	0.2 μM
Lucigenin	CC: 14872	Intracellular Chloride ion	Y	N (Y)	1 μM
CONA-Lectin	TFS: C21402	Binds α-man, α-glc	Y	Y (N)	2 μg/ml
EUA-Lectin	SA: L8262	Binds α-L-fuc and (glcNAc)2	Y	N (N)	2 μg/ml
PNA-Lectin	SA: L7381	Binds β-gal(1 → 3)galNAc	Y	Y (Y)	2 μg/ml
WGA-Lectin	TFS: W32465	Binds sialic acid and Nac	Y	Y (N)	2 μg/ml
pHrodo Red	TFS: P35372	Intracellular pH indicator	Y	Y (lables)	5 μM
LysoTracker Deep Red	TFS: L12492	Acidic organelles	Y	Y (lables)	0.2 μM
CellROX green	TFS: C10492	Reactive oxygen species	Y	Y (lables)	1 μM
Phen Green	TFS: P6763	Heavy metal indicator	Y	Y (lables)	1 μM
ALDAFLUOR	ST: 1700	ALDH-bright cells	Y	Y (lables)	0.4 μg/ml
DAPI	TFS: D1306	AT regions of DNA	Y	Y (Y)	3 μM
PI	TFS: R37108	DNA	Y	Y (N)	5 μg/ml

*P. damicornis* cell suspensions were incubated with indicated chemical compounds and analyzed by FACS. Companies used: *CC* Cayman Chemical, *TFS* Thermo Fisher Scientific, *SA* Sigma-Aldrich, *ST* Stemcell Technologies. If the chemical compound is fluorescently labeling cells it is indicated as Y (Yes) and if the result is the same as the control it is indicated as N (No) in the label column. If the labeling can clearly differentiate cellular populations by the level of labeling it is indicated as Y in the differentiation column of cells, differentiation capability of cells that are positive to *Symbiodinium* indicated in scopes. The concentration used throughout the study are indicated in the last column

For the phagocytosis assay, we used green colored beads called Fluoresbrite® YG Carboxylate Microspheres 1.00 μm (Polysciences), in a 2:1 beads:cells ratio. Sorted cells were incubated for 3 h or overnight at room temperature in staining media (10^5^cells/200 μl). The FACS reading was done either on BD FACS Aria II, BD LSRFortessa, BD FACS Accuri C6 (Becton, Dickinson Biosciences) or SH800S Cell Sorter (Sony). Analysis of flow cytometry data was done with FlowJo V10 program (FlowJo). The specific excitation laser and optical filter for emission measurements for each of the analysis is stated in the figures. The excitation laser was ﻿measured in nm and filters are stated as long pass (LP) and band pass (BP).

### Cell sorting using FACS and live cells microscopy

Cells were sorted by using the BD FACS Aria II, and gating was done using the BD FACSDiva™ software (Becton, Dickinson Biosciences). Cell sorting was performed with 100 μm nozzle size and sorted directly into 5 ml tubes containing 3 ml of staining media in order to minimize cellular stress. Cells (10,000–40,000) of each population of interest were sorted at a speed of 1500 cells/s.

For live cells microscopy, after washing in staining media by centrifugation at 4 °C; 500 x g; 5 min, the cells were resuspended in 30 μl of staining media and placed onto 18 well flat μ-slides coated with poly-L-lysine (Ibidi) and incubated overnight at 4 °C. Images of sorted cells were obtained using light microscopy.

## Results

### Cellular dissociation of coral cells and initial gating strategy

Dissociation of cells was accomplished using mechanical tools rather than enzymatic tools in order to prevent proteolysis of the membrane-bound proteins and to preserve cell viability. Using physical dissociation less cell death was observed, whereas, the use of the serine protease, trypsin, increased the percentage of dead cells in suspension by four-fold (Additional file 1: Figure S1).

**Fig. 1:**
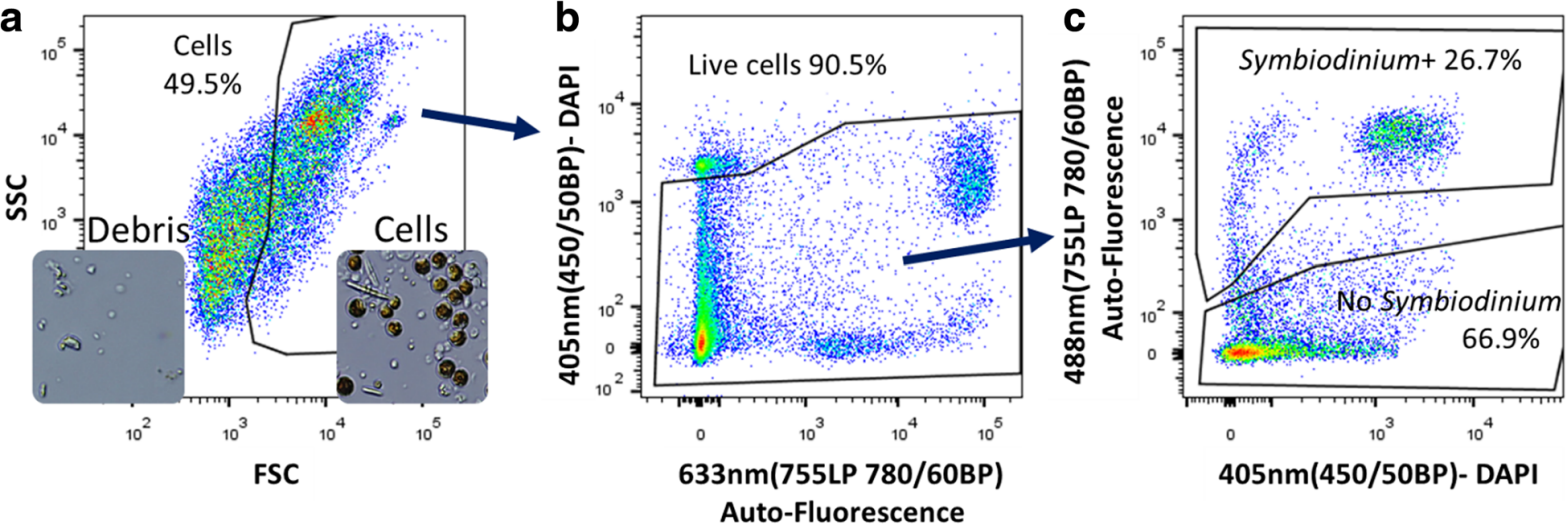
Coral cell gating strategy for population analysis by FACS. **a**) *P. damicornis* cell suspension analyzed using FACS ARIA II according to their intrinsic size (FSC) and granularity (SSC) properties on a log scale, exited by 488 nm laser. The gate separates cells from debris. Within the inserts are representative images of the sorted cells or debris populations. Blue scale bar = 20 μm. **b**) Gating on live cells out of the cell gate using 405 nm (450/50BP)- DAPI negative gate. The fluorescent gates are in two dimensions due to *P. damicornis ﻿an﻿﻿d﻿ Symbiodinium* natural auto-fluorescence. **c**) After excluding debris and dead cells the 488 nm (755LP 780/60BP) channel was used to measure *Symbiodinium* auto-fluorescent in this Far-Red channel to differentiate cells with or without *Symbiodinium*

Prior to the screening of fluorescent reagents, a gating strategy was developed to differentiate live cells. Using the size channel (FSC) and the granularity channel (SSC), we created a gate to differentiate cells and debris by sorting and observation by microscopy (Fig. 1a). Dying cells were stained with the DNA markers, DAPI or PI, to eliminate them from the analysis (Fig. 1b). Lastly, we were able to differentiate between coral cells containing *Symbiodinium* from the rest of the coral cells by utilizing the Far-Red channels to capture the natural fluorescence of *Symbiodinium* [53] (Fig. 1c). In addition to sorting and observation by microscopy, we validated the assumption that the Far-Red channel positive cells are due to *Symbiodinium*, by using two strains of *Aiptasia pallida,* a wild type strain with *Symbiodinium* and an aposymbiotic *A. pallida* (APO) strain that is raised for years without *Symbiodinium*. 15% of the wild type strain cells were positive in the Far-Red channel, whereas, the APO had a hundred-fold less cells in the Far-Red channel (Additional file 1: Figure S2). Several Far-Red channels can be excited by 488 nm, 561 nm, or 633 nm lasers, and are able to specifically detect the natural fluorescence of *Symbiodinium*, therefore acting as a strong marker for symbiont presence.

### Fluorescent cell markers for cellular differentiation

**Fig. 2:**
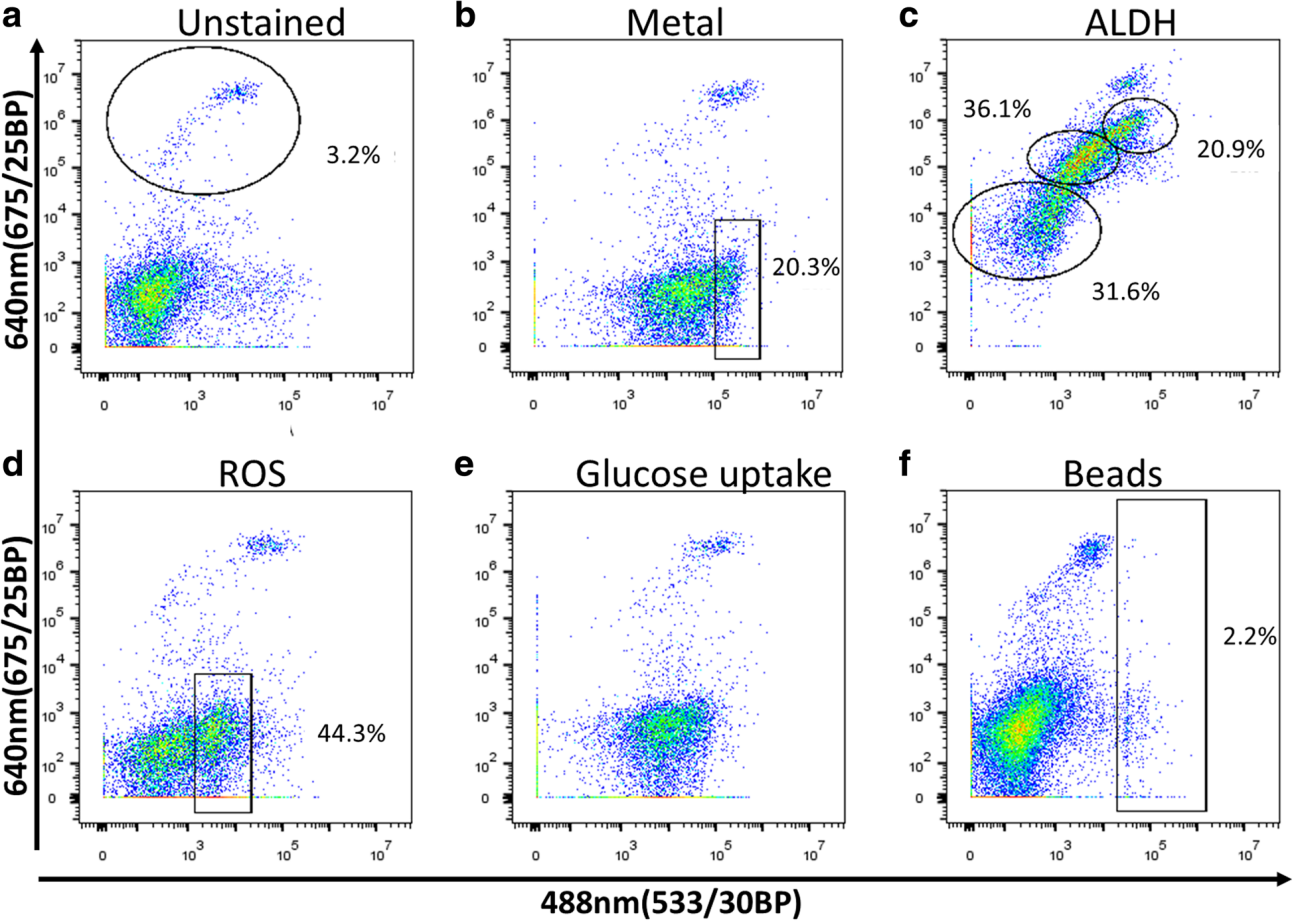
Coral cell differentiation patterns. *P. damicornis* cell suspension was incubated with different fluorescent markers and analyzed on FACS Accuri C6. All of the samples were analyzed on two dimensional plots of the green channel- 488 nm (533/30BP) and on the far-red channel-640 nm (675/25BP) to differentiate the *Symbiodinium* positive cells. Gated in black are the differentiated populations with their cell percentage indicated. The analysis was done after differentiation of cells and debris on FSC and SSC and live/dead cells using PI. **a**) Background control of unstained cells for all of the panels. In the gate are the *Symbiodinium* cells positive at 640 nm (675/25BP) due to natural fluorescence. **b**) Labeled cells with Phen Green, a color that is quenched by bi-valent metals. A small population of cells had the lowest level of metal and it differentiated from the main population. **c**) Labeled cells with ALDEFLUOR that indicates high levels of ALDH activity. No clear differentiation was found, but the width of the signal in different cells is 3 fluorescent log scales which enables us to differentiate low, moderate and high labeling. **d**) Cells containing ROS, labeled with CellRox. Clear negative and positive populations (positive marked) were identified. Note that all the *Symbiodinium* positive cells are positive to ROS. **e**) Cells were incubated with glucose analog 2-NBDG. Positive labeling was found, but no clear differentiation. **f**) Cells were co-incubated for 3 h with green fluorescent beads for analysis of engulfment. Cells positive to beads are marked

**Fig. 3:**
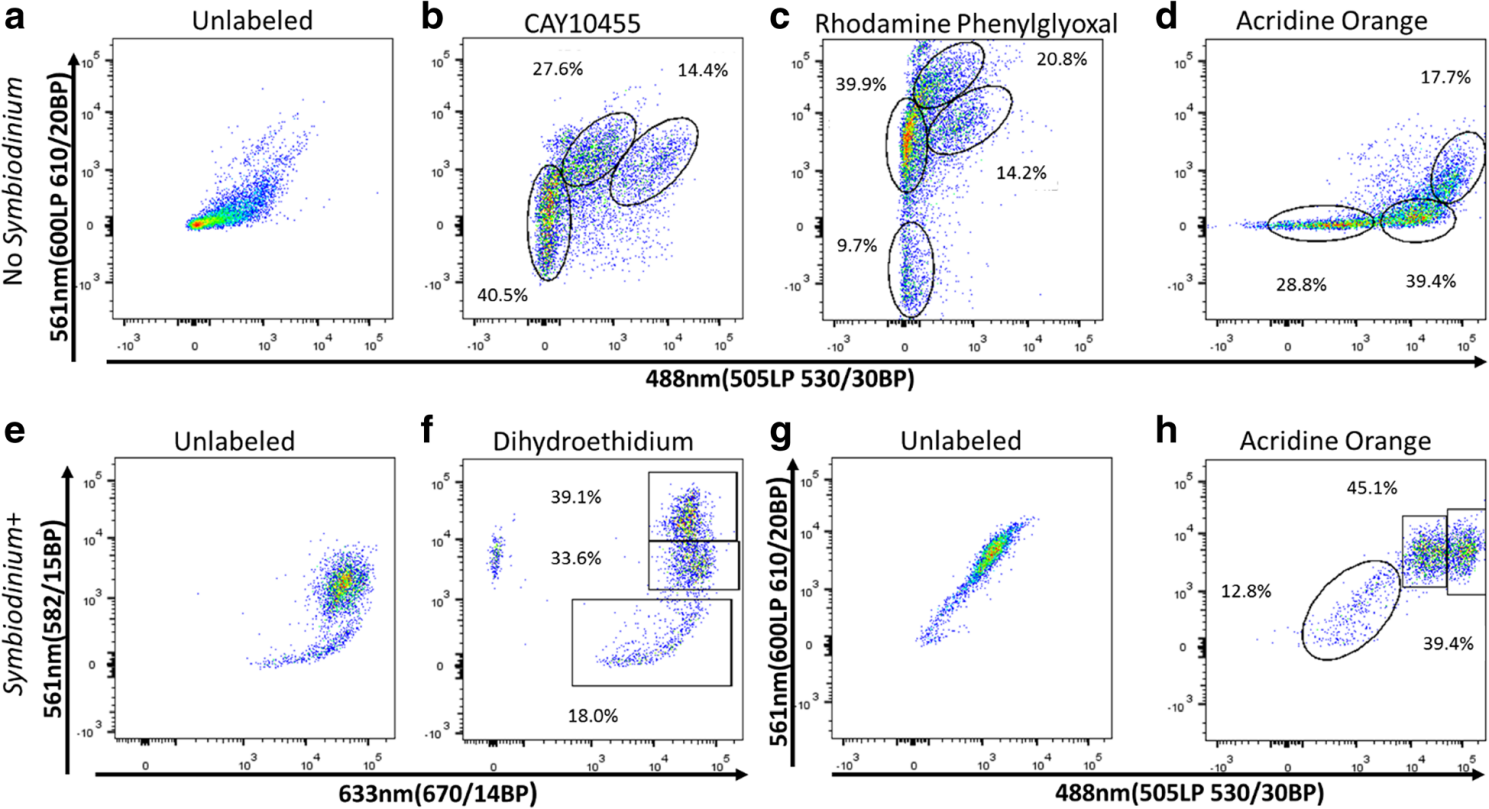
Examples of cell differentiation from a chemical compound screening. The *P. damicornis* cell suspension was incubated with the indicated chemical compounds for 30 min at 20^o^C and analyzed by FACS LSRFortessa. The analysis was done after differentiation of cells and debris on FSC and SSC and live/dead cells using DAPI, and separated to *Symbiodinium* positive cells (**e**–**h**) and *Symbiodinium* negative cells (**a**–**d**, as shown in Fig. 1). **a**) Background control of unstained *Symbiodinium* negative cells for (**b**–**d)**. **b**) Labeled cells with CAY10455, had separation for 3 potential populations shown in gates. **c**) Labeled cells with Rhodamine Phenylglyoxal had separation of 4 potential populations. **d**) Labeled cells with Acridine *Orange* had separation of 3 populations shown in red. **e**) Background control of unstained *Symbiodinium* positive cells for (**f)**. **f**) Labeled cells with Dihydroethidium had separation of 3 populations positive to *Symbiodinium*. **g**) Background control of unstained *Symbiodinium* positive cells for (**h**). **h**) Labeled cells with Acridine *Orange* had separation of 3 populations positive to *Symbiodinium*

After establishing the basic gating parameters, we sorted the cells further into cell type groups. We developed methods for detection of coral cell types and cell populations by FACS, using the fluorescence of the samples. We screened for non-species specific fluorescent markers, using i) fluorescent reagents that are enzymatically activated, ii) fluorescent molecules that mimic natural substrates, iii) detection of specific molecules in cells or vacuoles such as Phen-Green for bi-valent metals, iv) functional activity such as phagocytosis, and v) lectins for the staining of different glycosylation patterns on membrane bound proteins (Figs. 2, 3 and Table 1).

When screening for cell detection markers we looked for the following parameters: i) is there labeling of cells or not (positive staining compared to unstained control, Fig. 2b), ii) does the labeling enable cell population separation (Fig. 2d), and iii) if there is no separation, is there a wide dynamic range of fluorescence between high and low labeled cells that will distinguish different cell populations (Fig. 2c). For instance, labeling with Phen Green, which is used to identify metal ions, all the cells compared to the control were labeled, but a distinction between cell populations that express positive and those that express a higher absolute positive signal can be detected (marked in red, Fig. 2b). Another example is when using ALDEFLUOR to detect stem cells and pluripotent cells through the activity of aldehyde dehydrogenase (ALDH) [54]. Labeling with ALDEFLUOR does not differentiate populations, but because the labeling is so broad (wide dynamic range: 10^3^–10^6^ in 640 nm (675/25BP) fluorescence channel, and 10^2^–10^5^ in the 488 nm (533/30BP) fluorescence channel) gates can be made to distinguish between low, moderate, and high fluorescent labeling (marked in red, Fig. 2c).

In the testing for reactive oxygen species (ROS) using CellROX and phagocytosis using microbeads, we found a clear positive population compared to the rest of the cells (gated in Fig. 2d, f). This indicates that there are populations of cells that are acting similar to immune cells and can be detected by immune markers. Glucose analog uptake measured by 2-NBDG, which is an indicator of the energetic state of the cells, is positive in all cells compared to unstained samples, but it does not give a clear signal of cell separation (Fig. 2e). Due to this reason the reagent was not a good candidate for identifying different cell types. Lastly, cells containing *Symbiodinium* can be differentiated by using the Far-Red [640 nm (675/25BP) channel (gated in Fig. 2a and Additional file 1: Figure S2)], and also together with the screened markers as seen in all panels of Fig. 2 in the Far-Red [640 nm (675/25BP)] channel.

**Fig. 4:**
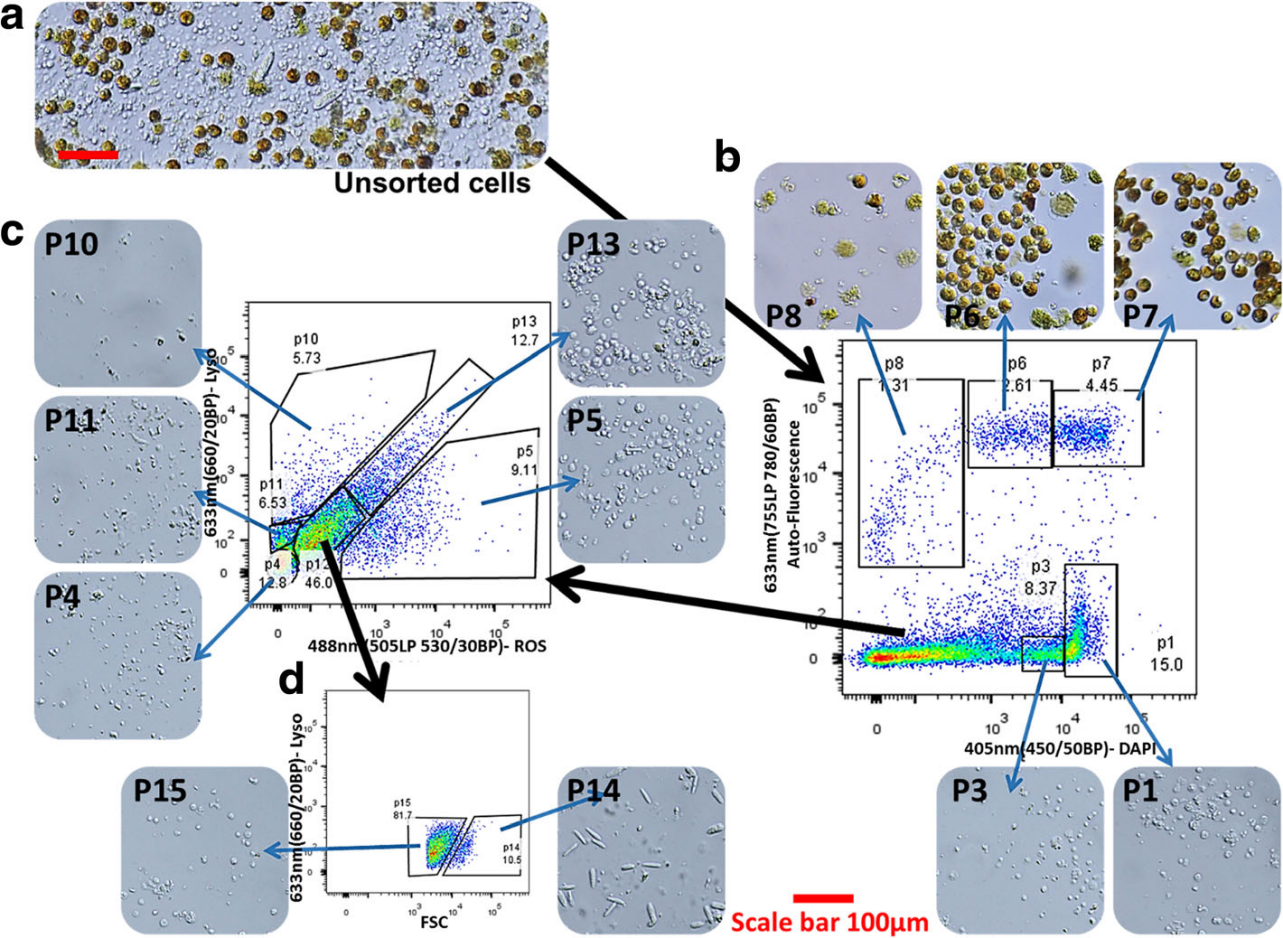
12 coral cell populations sorted by FACS. *P. damicornis* cell suspension was labeled with DAPI, Cellrox, and LysoTracker Deep Red, analyzed and sorted on FACS ARIA II. Sorted cells were put on *ibidi* 18 microwell plate coated with poly-L-lysine, overnight at 4^o^C prior to obtaining the cell images by light microscopy (image inserts). **a**) Light microscopy image of unsorted cells. **b**) After excluding cell debris by their intrinsic size (FSC) and granularity (SSC), we gated a two dimensional plot of the 633 nm (755LP 780/60BP) channel containing the *Symbiodinium* positive cells and DAPI stained cells in the 405 nm (450/50BP) channel. We sorted 3 *Symbiodinium* positive populations (upper panels, p6–8), and two positive populations for DAPI (lower inserts, p3 and p1). **c**) Ungated cells in panel b were analyzed on two dimensional plot of 488 nm (505LP 530/30BP) channel- ROS and 633 nm (660/20BP) channel- LysoTracker. An additional 6 populations were differentiated by this panel (p4–5 and p10–13). **d**) Population 12 (P12) was additionally separated into two populations on panel of size (FSC) and LysoTracker (APC) to P14 and P15. Notably, P14 is enriched for nematocyst cells. Red scale bar for all inserts = 100 μm

We also screened 23 different fluorescent chemical compounds for detecting coral cell populations (Cayman Chemical). These fluorescent markers are associated with specific cellular activity, including enzymatic activity, elastase activity, cytochrome P450 activity, and indicators of zinc, calcium, and dsRNA. It is important to mention that these fluorescent markers do not have the same classical excitation and emission wavelengths of the majority of widely used FACS markers, but rather give different labeling patterns in different channels (Fig. 3b and c). For instance, Rhodamine Phenylglyoxal has different emission patterns in the 561 nm channel (600LP 610/20BP) and 488 nm channel (505LP 530/30BP) even though they are both excited by the 488 nm laser. This different pattern of emission enables us to differentiate at least 4 cell populations using a single reagent in a two-dimension analysis (Fig. 3c). We analyzed these 23 different fluorescent reagents in the 561 nm channel (582/15BP), the 561 nm channel (600LP 610/20BP), the 633 nm channel (670/14BP), and the 488 nm (505LP 530/30BP), and used DAPI to detect live/dead cells. *Symbiodinium* positive cells were analyzed using the Far-Red channe l561 nm (755LP 780/60BP) while the fluorescence of screened markers was checked in Far-Red channel 633 nm (670/14BP) (Fig. 3 on BD LSRFortessa machine). Thiscan be accomplished on any machine that has at least two channels in Far- Red, such as APC, AF700, PE-APC, PE-TexasRed and APC-Cy7 (Fig. 4 on BD FACS Aria II machine). This can be done because the natural fluorescence of *Symbiodinium* will have the same fluorescence pattern in all of these channels. In addition, this can also be done within a single channel of Far-Red if the labeling at 640 nm (675/25BP) is lower than the *Symbiodinium* natural fluorescence (Fig. 2c on BD Accuri C6 machine). By using these techniques, we revealed that different populations of *Symbiodinium* positive cells can be defined, using channels such as ThioFluor623, Lucigenin, and Zinpyr-1 (Fig. 3e–h). Lastly, Acridine Orange can differentiate three populations of *Symbiodinium* positive cells, and three from the rest of the cells types, giving at least six cell populations within a single labeling (Fig. 3d and h). The results of all the fluorescent markers used in this study are summarized in Table 1. In total our screening procedure of Cayman Chemical compounds resulted in 15 compounds that labeled coral cells, ﻿with ﻿10 of these compounds able to differentiate cell populations.

### Use of fluorescent cell markers for cell population sorting

We validated morphologically that we could sort different cell populations using the screened fluorescent markers. We then used a panel of three dyes (DAPI, Cellrox, and LysoTracker Deep Red) with a different channels gating strategy and sorted 12 coral cell populations (Fig. 4). The sorted cells were imaged in order to assess the morphology and homology within each sorted population (Image inserts in Fig. 4). Based on this panel, we succeeded in separating three populations of *Symbiodinium* positive cells that look very different at a morphological level (Fig. 4). In addition, 9 populations were sorted, which looked relatively homogeneous. For example, ﻿population 14 (P14) is enriched for cnidocytes, the stinging cells of cnidarians (Fig. 4d). This validated the assumption that the cells we sorted using our FACS differentiation markers and gating strategy were different cell populations on the morphological level.

Corals have two tissue layers, an outer epidermis and an internal gastrodermis. These layers are separated by a mesoglea layer, a primitive type of mesoderm, that is made of connective tissue, and has few cells present including secretory cells, amoeboid cells, and reproductive cells [39]. Many cell types reside within the epidermis, including ciliated column cells, secretory cells, sensory motor neuron cells, interneuron cells, neurosecretory cells, sensory cells, cnidocytes, and flagellated columnar cells [39]. In the gastrodermis, cell types include cuboidal cells, absorptive cells, secretory cells, squamous cells, columnar cells, anchoring cells, flagellated columnar cells, flagellated cuboidal cells, spindle shaped cells, sensory cells, motor neurons, interneurons, neurosecretory and *Symbiodinium* (algal symbiotic cells which live within gastrodermis cells) [39]. Overall, in Fig. 4, we can see many of these previously mentioned cell types, including the cnidocytes in population P14, and *Symbiodinium* in populations P6, P7, and P8. The other cell populations appear to be a mix of various cells from each layer, but have similar fluorescent outputs, and therefore partitioned into the same population. Based on morphological observations, populations P13, and P5 appear to have very similar cell types present, including round granular type cells, and some rod shaped cells. Population P10 and P4 are also very similar; both have irregular shaped, smaller cell types. Population P15 has several cell types that have a granular appearance, as does population P3 and P1. Further investigation will need to be explored to characterize these cell types in this isolated form, and assign them to their tissue layer. One possibility is using the same labels with fluorescent microscopy on an intact specimen to try to understand the localization of the cells within the animal.

### Validation of the fluorescent cell markers on symbiotic anemone

**Fig. 5:**
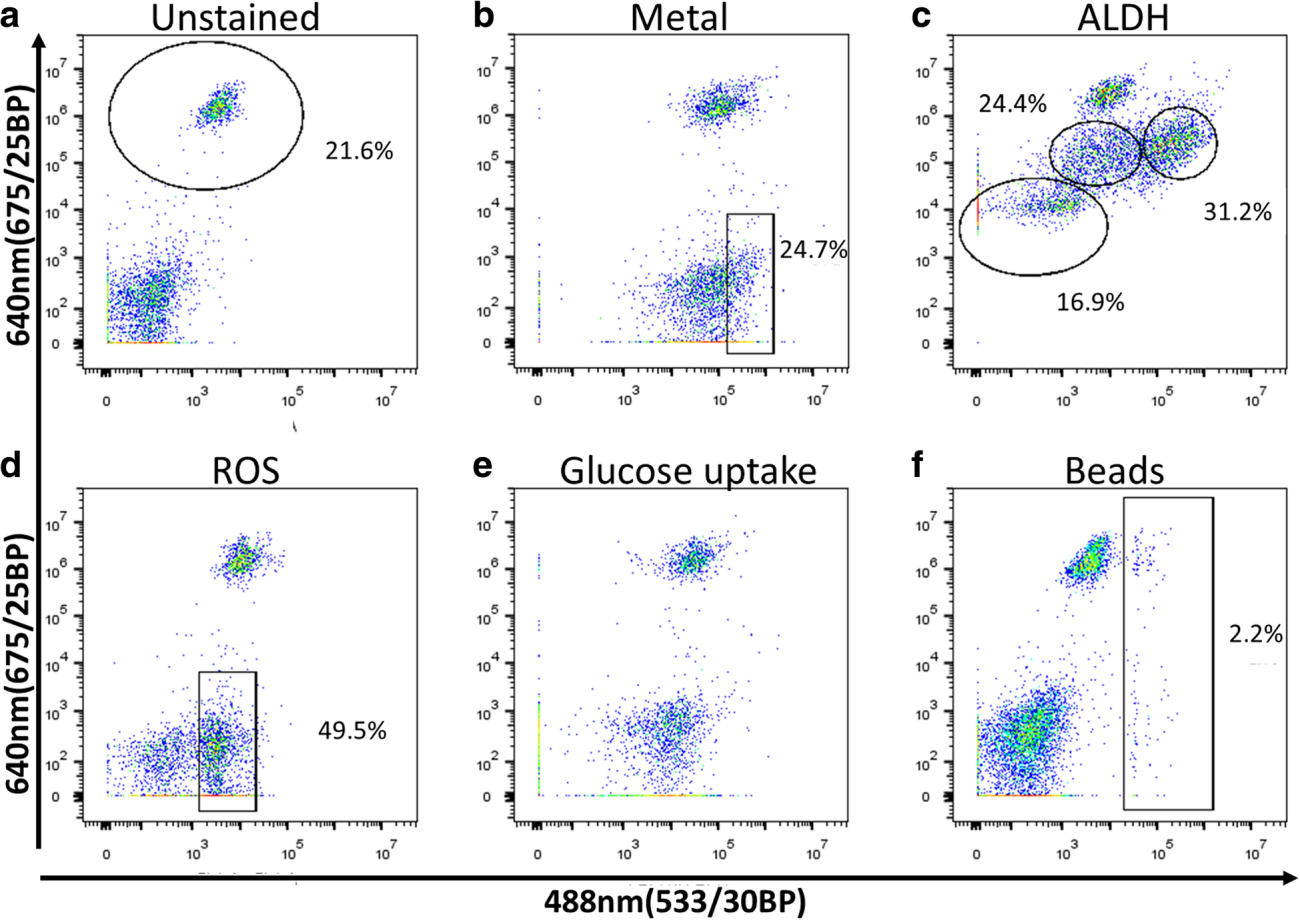
*A. pallida* cells labeled with fluorescent markers. The *A. pallida* cell suspension was incubated with the same fluorescent markers that were used to label coral cells and analyzed on FACS AccuriC6. All the samples were analyzed on two dimensional plots of the 488 nm channel (533/30BP) which detec﻿ts the fluorescent markers and on the Far-red 640 nm channel  (675/25BP) which differentiates the *Symbiodinium* positive cells. Marked in the gates are the differentiated populations. The analysis was done after differentiation of cells and debris on FSC and SSC, and live/dead cells using PI. **a**) Background control of unstained cells in the gate are the *Symbiodinium* cells positive in the 640 nm channel (675/25BP) due to natural fluorescence. **b**) Labeled cells with Phen Green, a color that is quenched by bi-valent metals. A small population of cells with the lowest level of metal was found to differentiate from the main population, labeled in the gate. **c**) Labeled cells with ALDEFLUOR a marker for ALDH activity. In the gate are low, moderate and high labeling populations. **d**) Cells labeled with CellRox, a label for ROS. In this case negative and positive populations are detected (positive marked). **e**) Cells incubated with glucose analog 2-NBDG. The cells are positive, but no clear differentiation. **f**) Cells co-incubated for 3 h with green fluorescent beads for analysis of phagocytosis. Cells positive for bead phagocytosis are gated

We tested the translatability of our work with non-specific markers on other related species by examining *A. pallida* (Order: Actiniaria), a sea anemone that harbors *Symbiodinium* similar to corals. With only minor changes to the level of the fluorescence labeling, the same reagents that were used previously for the coral *P. damicornis* exhibited the similar cell labeling and cell separation patterns (Fig. 5). This included the differentiation of *Symbiodinium* from the rest of the cells, and multiple cell populations as in the case of ALDH (Fig. 5a and c). This result validates our previous work with *P. damicornis.* Additionally, the use of the screened reagents and the gating strategy we have developed shows promise for use in multiple species of cnidarians.

### Validation of down-stream process on sorted cells based on lysolitic vesicles

**Fig. 6:**
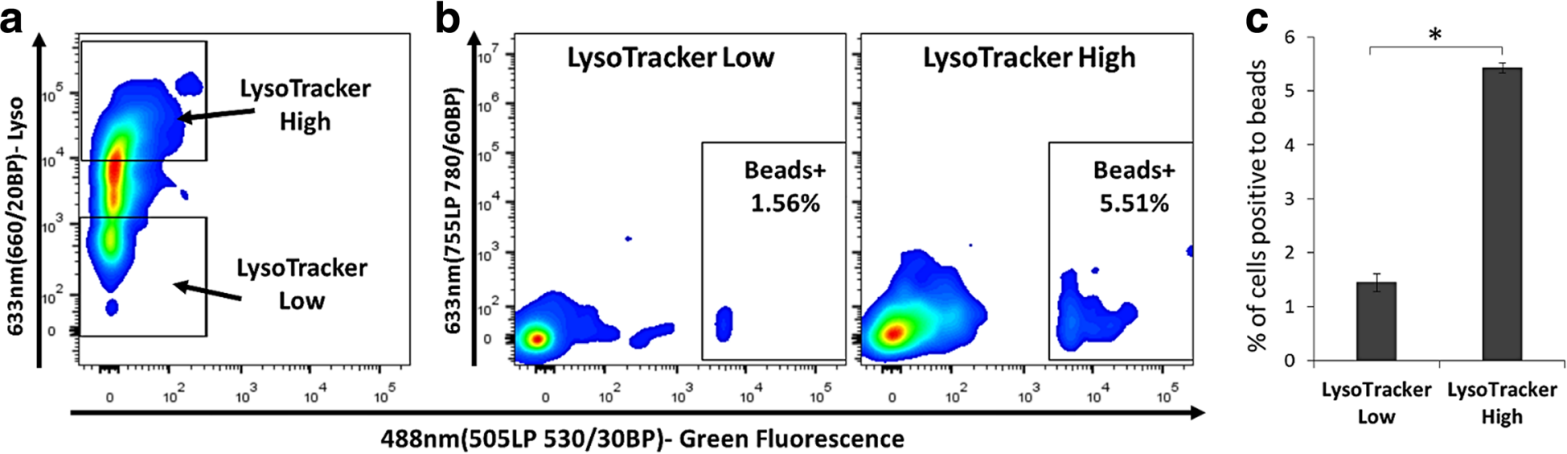
Downstream process validation using a phagocytosis assay. **a** *A. pallida* cells were labeled with LysoTracker Deep Red and sorted to LysoTracker high and low expression. **b**–**c** The sorted cells were co-incubated overnight with green fluorescent beads for analysis of p﻿﻿hagocytosis. **b** Example of the bead positive cells gated in the LysoTracker low (left panel) and high (right panel) populations. **c** Analysis of bead positive cells of three replicates of the sorted populations. The bead positive cells are approximately three - fold higher in the LysoTracker high population. ANOVA P* < 0.05

In order to validate dow﻿n-stream processes on sorted cells by FACS, we performed an immune-associated functional assay of phagocytosis. For sorting, we used LysoTracker, and tested its association with phagocytic cells. Phagocytic cells  should contain phagolysosomes and lysosomes in higher level than other cell populations. We labeled *A. pallida* cells with LysoTracker Deep Red and sorted cells that did not contain *Symbiodinium*, into LysoTracker high and low labeling (Fig. 6a). After overnight incubation with fluorescent beads, the LysoTracker high population had approximately four-fold higher positive cells containing beads than the low population (Fig. 6b and c). To validate this finding, we ran the beads alone and found that due to their size (1 μm), the amount of beads that could pass the gate of the cells is neglectable (Additional file 1: Figure S3). This experiment shows two main findings: 1) sorted cells can be used for downstream processes that need live cells for functionality, like phagocytosis and 2) demonstrates that we can use not only non-﻿species specific markers, but also physiological and funcational markers for additional characterization of the cells.

## Discussion

### Practical uses for live coral cell separation

In the current study we used mechanical dissociation, and discerned between live and dead cells and debris using FSC, SSC and DNA dyes. We then screened for different fluorescent markers to isolate and sort coral cell populations. We used a diverse arra﻿y of flow cytometers for our analysis in order to show that this method can be done on variety of FACS instruments, from machines with 4 channels and 2 lasers (BD Accuri C6; Figs. 2 and 5) to machines with 14 channels and 4 lasers (BD LSRFortessa; Fig. 3). We also developed cell isolation methods for coral tissue, and identified conditions in which cells can be cultured* ex-vivo*. Lastly, we assayed for *ex-vivo* cellular function, such as phagocytosis and glucose uptake (Figs. 2, 5 and 6).

These methods have many important down-stream applications, including cell culture, cell specific functional assays, RNA-sequencing, immune assays, transplantation, as well as gene and protein expression done in mammalian and non-mammialian animal models [43–45, 48, 49, 51]. As a proof of concept, we performed phagocytosis assays on sorted cell populations (Fig. 6). Utilizing this technique in corals will allow for a better understanding of different coral cells types, and will be useful in understanding how different cell populations react to different stressors, including heat-induced stress, immune challenges, endosymbiosis processes, and acidification treatments, In total, we screened over 30 different cell markers, which can be used to identify different cellular populations. In the future, a combination of our described FACS methods using the same reagents for fluorescent microscopy in a gross animal specimen could reveal localization of specific cell populations in-vivo.

### Identification of different coral cell populations

We examined the use of fluorescent markers to separate coral cell populations. From this, we identified 12 different populations including three that include *Symbiodinium* (Fig. 4). We have shown that we can use a combination of screened dyes to sort large numbers of cellular populations with different morphologies. We would like to emphasize that we separated cells based on three non-species specific markers. Combinations of other markers, or the addition of specific antibodies, will lead to a greater degree of population separation. The markers that we have used are associated with a specific enzymatic or cellular activity, a compound, or an organelle, and therefore are indicators of biological function. For example, cells positive for ROS and LysoTracker (Population 13 in Fig. 4) could be immune-like phagocytic cells; we would expect this type of cell to have lysolitic vesicles containing ROS compounds [55–58]. These cells could be targeted for future research using beads and bacteria engulfment to demonstrate immune-like phagocytosis. In *A. pallida*, we have successfully shown that LysoTracker positive cells are associated with higher phagocytosis level compared to LysoTracker low cells (Fig. 6).

The ALDH high population may also prove to be a population of interest (Fig. 2c, gated in upper right side). High ALDH activity is associated with stem and progenitor cells [54] and can enrich stem cells in invertebrates [49]. Isolation of these cells would be valuable for understanding the regenerative capabilities of corals and could lead to a better understanding of wound healing processes.

Lastly, we found markers that did not clearly label different cell populations, such as the glucose uptake marker 2-NBDG (Fig. 2e). While it is not a good marker for cell separation, it is a good candidate for assaying glucose uptake in corals in experimental settings such as in response to heat stress. Previous studies have shown that glucose and glycerol are translocated from* Symbiodinium* to the coral host [59]. These critical metabolites are important for the coral overall health and low levels could indicate a nutritional deficit for the coral host.

While the majority of the markers used today in flow cytometry are antigen-specific, we have used no﻿n-species specific fluorescent labeling compounds that can be easily translated to non-model organisms. As a proof of concept, we performed assays on two different cnidarians and found labeling patterns to be very similar (Fig. 5 compared to Fig. 2). Therefore, it is likely that this method could be used on other cnidarian species. Corals express many different fluorescent proteins and there is variation in expression between different corals [60]. Therefore, minor adjustments may be necessary in developing a gating strategy for the successful separation in these populations. With the validation of this technique in both *A. pallida* and *P. damicornis*, we are confident that this strategy will be valuable for studies in other cnidarians as well.

## Conclusions

In this study, we adapted the FACS method for studying coral cells. We developed a gating strategy for identifying 12 separate coral cell populations and tested over 30 different fluorescent markers. We verified this in another cnidarian, *A. pallida,* and found that a similar strategy can be used, indicating the utility of this technique in other cnidarians. We have shown that sorted cells can be used in down-stream live functional assays and that the markers used provide valuable insights into the biology of coral cells. This technique will be extremely valuable for studying the cellular role of stress response, as well as for immunological studies in corals and other cnidarians.

## Additional file

Additional file 1: Figure S1. Comparison of coral cell dissociation. *P. damicornis* cell suspension was collected either mechanically using a fine blade as described in the methods (A) or also incubated at room temperature with Trypsin 0.025% of Trypsin-EDTA in 3.3XPBS (B). Cell suspension was labeled with DAPI, CellRox, and LysoTracker Deep Red, and analyzed using SH800S Cell Sorter. Among the cells isolated without Trypsin (A) about 8% were positive to DAPI whereas 31% were positive to DAPI isolated with Trypsin (B), in the rectangular gate. This DAPI stain results corroborates our observation with Trypan Blue by light microscopy (data not shown). **Figure S2.** *Symbiodinium* positive population validation. In addition to the sorted cells microscopy, to validate that the population observed on the far red and green channels without labeling is *Symbiodinium* we used wild type (WT) *Aiptasia* that contains *Symbiodinium* and stable strains that stably grow for more than a decade without any symbiotic algae (APO). It can be observed that the gate of *Symbiodinium* positive cells is almost empty of cells (Right panel; 0.17%) in the APO strain while there are about 15% of cells in the gate of the wild type strain. **Figure S3.** Green fluorescent beads control for phagocytosis assays. To validate that the green fluorescent beads positive cells are the gated populations (Figs. 2, 5, 6) we ran the beads alone. Due to the size of the beads (1 μm) most of them that are not conjugated to cells are gated out on the size and granularity gate (FSC, SSC). Only 8 out of 10,000 events are positive in the gate. (DOCX 577 kb)

## References

[1] Hughes TP, Rodrigues MJ, Bellwood DR, Ceccarelli D, Hoegh-Guldberg O, McCook L, Moltschaniwskyj N, Pratchett MS, Steneck RS, Willis B (2007). Phase shifts, herbivory, and the resilience of coral reefs to climate change. Curr Biol.

[2] Bellwood DR, Hughes TP (2001). Regional-scale assembly rules and biodiversity of coral reefs. Science.

[3] Hoegh-Guldberg O, Mumby PJ, Hooten AJ, Steneck RS, Greenfield P, Gomez E, Harvell CD, Sale PF, Edwards AJ, Caldeira K (2007). Coral reefs under rapid climate change and ocean acidification. Science.

[4] Hoegh-Guldberg O, Bruno JF (2010). The impact of climate change on the world's marine ecosystems. Science.

[5] Hughes TP, Kerry JT, Alvarez-Noriega M, Alvarez-Romero JG, Anderson KD, Baird AH, Babcock RC, Beger M, Bellwood DR, Berkelmans R (2017). Global warming and recurrent mass bleaching of corals. Nature.

[6] Schwarz JA, Krupp DA, Weis VM (1999). Late larval development and onset of Symbiosis in the Scleractinian coral Fungia Scutaria. Biol Bull.

[7] Rodriguez-Lanetty M, Wood-Charlson EM, Hollingsworth LL, Krupp DA, Weis VM (2006). Temporal and spatial infection dynamics indicate recognition events in the early hours of a dinoflagellate/coral symbiosis. Mar Biol.

[8] Bieri T, Onishi M, Xiang TT, Grossman AR, Pringle JR. Relative Contributions of Various Cellular Mechanisms to Loss of Algae during Cnidarian Bleaching. PLoS One. 2016;11(4):1–24. 10.1371/journal.pone.0152693 PMC4847765 27119147

[9] Traylor-Knowles N, Rose NH, Palumbi SR (2017). The cell specificity of gene expression in the response to heat stress in corals. J Exp Biol.

[10] Gates RD, Baghdasarian G, Muscatine L (1992). Temperature stress causes host-cell detachment in symbiotic cnidarians - implications for coral bleaching. Biol Bull.

[11] Perez S, Weis V (2006). Nitric oxide and cnidarian bleaching: an eviction notice mediates breakdown of a symbiosis. J Exp Biol.

[12] Sawyer SJ, Muscatine L (2001). Cellular mechanisms underlying temperature-induced bleaching in the tropical sea anemone Aiptasia Pulchella. J Exp Biol.

[13] Ainsworth TD, Hoegh-Guldberg O (2008). Cellular processes of bleaching in the Mediterranean coral Oculina Patagonica. Coral Reefs.

[14] Brown BE, Letissier MDA, Bythell JC (1995). Mechanisms of bleaching deduced from histological studies of reef corals sampled during a natural bleaching event. Mar Biol.

[15] Dunn SR, Thomason JC, Le Tissier MDA, Bythell JC (2004). Heat stress induces different forms of cell death in sea anemones and their endosymbiotic algae depending on temperature and duration. Cell Death Differ.

[16] Vidal-Dupiol J, Adjeroud M, Roger E, Foure L, Duval D, Mone Y, Ferrier-Pages C, Tambutte E, Tambutte S, Zoccola D (2009). Coral bleaching under thermal stress: putative involvement of host/symbiont recognition mechanisms. BMC Physiol.

[17] Domart-Coulon IJ, Elbert DC, Scully EP, Calimlim PS, Ostrander GK (2001). Aragonite crystallization in primary cell cultures of multicellular isolates from a hard coral, Pocillopora Damicornis. Proc Natl Acad Sci U S A.

[18] Domart-Coulon IJ, Sinclair CS, Hill RT, Tambutte S, Puverel S, Ostrander GK (2004). A basidiomycete isolated fromt eh skeleton of Pocillopora Damicornis (Scleractinia) selectively stimulates short-term survival of coral skeletogenic cells. Mar Biol.

[19] Puverel S, Tambutte E, Zoccola D, Domart-Coulon I, Bouchot A, Lotto S, Allemand D, Tambutte S (2005). Antibodies against the organic matrix in scleractinians: a new tool to study coral biomineralization. Coral Reefs.

[20] Moya A, Tambutte S, Bertucci A, Tambutte E, Lotto S, Vullo D, Supuran CT, Allemand D, Zoccola D (2008). Carbonic anhydrase in the scleractinian coral Stylophora Pistillata - characterization, localization, and role in biomineralization. J Biol Chem.

[21] Venn A, Tambutte E, Holcomb M, Allemand D, Tambutte S. Live Tissue Imaging Shows Reef Corals Elevate pH under Their Calcifying Tissue Relative to Seawater. PLoS One. 2011;6(5):1–9. 10.1371/journal.pone.0020013 PMC3103511 21637757

[22] Zoccola D, Tambutte E, Kulhanek E, Puverel S, Scimeca JC, Allemand D, Tambutte S (2004). Molecular cloning and localization of a PMCA P-type calcium ATPase from the coral Stylophora Pistillata. Bba-Biomembranes.

[23] Bertucci A, Tambutte S, Supuran CT, Allemand D, Zoccola D (2011). A new coral carbonic anhydrase in Stylophora Pistillata. Mar Biotechnol.

[24] Zoccola D, Moya A, Beranger GE, Tambutte E, Allemand D, Carle GF, Tambutte S (2009). Specific expression of BMP2/4 ortholog in biomineralizing tissues of corals and action on mouse BMP receptor. Mar Biotechnol (NY).

[25] Zoccola D, Ganot P, Bertucci A, Caminiti-Segonds N, Techer N, Voolstra CR, Aranda M, Tambutte E, Allemand D, Casey JR (2015). Bicarbonate transporters in corals point towards a key step in the evolution of cnidarian calcification. Sci Rep.

[26] Domart-Coulon I, Ostrander GK, Woodley CM, Downs CA, Bruckner AW, Porter JW, Galloway SB (2015). Coral cell and tissue culture methods. Diseases of corals.

[27] Domart-Coulon I, Tambutte S, Tambutte E, Allemand D (2004). Short term viability of soft tissue detached from the skeleton of reef-building corals. J Exp Mar Biol Ecol.

[28] Lecointe A, Cohen S, Geze M, Djediat C, Meibom A, Domart-Coulon I (2013). Scleractinian coral cell proliferation is reduced in primary culture of suspended multicellular aggregates compared to polyps. Cytotechnology.

[29] Lecointe A, Geze M, Djediat S, Domart-Coulon I. Coral cell proliferation in situ (polyp) and in primary cultures (3D aggregates). Cytotechnology. 2013;65(5) 679–679 10.1007/s10616-013-9562-6 PMC3967603 23756729

[30] Venn AA, Tambutte E, Lotto S, Zoccola D, Allemand D, Tambutte S (2009). Imaging intracellular pH in a reef coral and symbiotic anemone. Proc Natl Acad Sci U S A.

[31] Gibbin EM, Putnam HM, Davy SK, Gates RD (2014). Intracellular pH and its response to CO2-driven seawater acidification in symbiotic versus non-symbiotic coral cells. J Exp Biol.

[32] Barott KL, Perez SO, Linsmayer LB, Tresguerres M (2015). Differential localization of ion transporters suggests distinct cellular mechanisms for calcification and photosynthesis between two coral species. Am J Physiol-Reg I.

[33] Quistad SD, Stotland A, Barott KL, Smurthwaite CA, Hilton BJ, Grasis JA, Wolkowicz R, Rohwer FL (2014). Evolution of TNF-induced apoptosis reveals 550 my of functional conservation. Proc Natl Acad Sci U S A.

[34] Krediet CJ, DeNofrio JC, Caruso C, Burriesci MS, Cella K, Pringle JR. Rapid, Precise, and Accurate Counts of Symbiodinium Cells Using the Guava Flow Cytometer, and a Comparison to Other Methods. PLoS One. 2015;10(8):1–19. 10.1371/journal.pone.0135725 PMC4546242 26291447

[35] Mass T, Drake JL, Peters EC, Jiang W, Falkowski PG (2014). Immunolocalization of skeletal matrix proteins in tissue and mineral of the coral Stylophora Pistillata. Proc Natl Acad Sci U S A.

[36] Peters EC, Halas JC, McCarty B (1985). Calicoblastic neoplasms in Acropora Palmata, with a review of reports on anomalies of growth and form in corals. J Natl Cancer Inst.

[37] Olano CT, Bigger CH (2000). Phagocytic activities of the gorgonian coral Swiftia Exserta. J Invertebr Pathol.

[38] Palmer CV, Traylor-Knowles NG, Willis BL, Bythell JC. Corals Use Similar Immune Cells and Wound-Healing Processes as Those of Higher Organisms. PLoS One. 2011;6(8) 10.1371/journal.pone.0023992 PMC3161096 21887359

[39] Peters EC, Woodley CM, Downs CA, Bruckner AW, Porter JW, Galloway SB (2016). Diseases of corals. Diseases of corals.

[40] Ainsworth TD, Krause L, Bridge T, Torda G, Raina JB, Zakrzewski M, Gates RD, Padilla-Gamino JL, Spalding HL, Smith C (2015). The coral core microbiome identifies rare bacterial taxa as ubiquitous endosymbionts. ISME J.

[41] Pei R, Lee JH, Shih NJ, Chen M, Terasaki PI (2003). Single human leukocyte antigen flow cytometry beads for accurate identification of human leukocyte antigen antibody specificities. Transplantation.

[42] Herzenberg LA, Parks D, Sahaf B, Perez O, Roederer M, Herzenberg LA (2002). The history and future of the fluorescence activated cell sorter and flow cytometry: a view from Stanford. Clin Chem.

[43] Laerum OD, Farsund T (1981). Clinical-application of flow-cytometry - a review. Cytometry.

[44] Carroll S, Al-Rubeai M (2004). The selection of high-producing cell lines using flow cytometry and cell sorting. Expert Opin Biol Ther.

[45] Edri-Brami M, Rosental B, Hayoun D, Welt M, Rosen H, Wirguin I, Nefussy B, Drory VE, Porgador A, Lichtenstein RG. Glycans in Sera of Amyotrophic Lateral Sclerosis Patients and Their Role in Killing Neuronal Cells. PLoS One. 2012;7(5):1–15. 10.1371/journal.pone.0035772 PMC3364259 22666317

[46] Rosental B, Shemesh A, Yaron-Mendelson M, Klein LC, Kodman Y, Levy J, Porgador A, Broides A (2015). Evaluation of purified natural killer cell functions in familial hemophagocytic lymphohistiocytosis. LymphoSign Journal.

[47] Rosental B, Hadad U, Sinay R, Braiman A, Porgador A, Pollack Y (2012). Dual fluorescent labelling of the human malaria parasite plasmodium falciparum for the analysis of the ABC type transporter pfmdr2. Malar J.

[48] Corey DM, Rosental B, Kowarsky M, Sinha R, Ishizuka KJ, Palmeri KJ, Quake SR, Voskoboynik A, Weissman IL (2016). Developmental cell death programs license cytotoxic cells to eliminate histocompatible partners. Proc Natl Acad Sci U S A.

[49] Laird DJ, De Tomaso AW, Weissman IL (2005). Stem cells are units of natural selection in a colonial ascidian. Cell.

[50] Allam B, Ashton-Alcox KA, Ford SE (2002). Flow cytometric comparison of haemocytes from three species of bivalve molluscs. Fish Shellfish Immunol.

[51] Pancer Z, Amemiya CT, Ehrhardt GR, Ceitlin J, Gartland GL, Cooper MD (2004). Somatic diversification of variable lymphocyte receptors in the agnathan sea lamprey. Nature.

[52] Lehnert EM, Burriesci MS, Pringle JR (2012). Developing the anemone Aiptasia as a tractable model for cnidarian-dinoflagellate symbiosis: the transcriptome of aposymbiotic A pallida. BMC Genomics.

[53] Davy SK, Allemand D, Weis VM (2012). Cell biology of cnidarian-dinoflagellate symbiosis. Microbiol Mol Biol Rev.

[54] Storms RW, Trujillo AP, Springer JB, Shah L, Colvin OM, Ludeman SM, Smith C (1999). Isolation of primitive human hematopoietic progenitors on the basis of aldehyde dehydrogenase activity. Proc Natl Acad Sci U S A.

[55] Daigneault M, Preston JA, Marriott HM, Whyte MK, Dockrell DH (2010). The identification of markers of macrophage differentiation in PMA-stimulated THP-1 cells and monocyte-derived macrophages. PLoS One.

[56] Garg S, Sharma M, Ung C, Tuli A, Barral DC, Hava DL, Veerapen N, Besra GS, Hacohen N, Brenner MB (2011). Lysosomal trafficking, antigen presentation, and microbial killing are controlled by the Arf-like GTPase Arl8b. Immunity.

[57] Arsenijevic D, Onuma H, Pecqueur C, Raimbault S, Manning BS, Miroux B, Couplan E, Alves-Guerra MC, Goubern M, Surwit R (2000). Disruption of the uncoupling protein-2 gene in mice reveals a role in immunity and reactive oxygen species production. Nat Genet.

[58] Forman HJ, Torres M (2002). Reactive oxygen species and cell signaling respiratory burst in mscrophage signaling. Am J Respir Crit Care Med.

[59] Burriesci MS, Raab TK, Pringle JR (2012). Evidence that glucose is the major transferred metabolite in dinoflagellate-cnidarian symbiosis. J Exp Biol.

[60] Alieva NO, Konzen KA, Field SF, Meleshkevitch EA, Hunt ME, Beltran-Ramirez V, Miller DJ, Wiedenmann J, Salih A, Matz MV. Diversity and Evolution of Coral Fluorescent Proteins. PLoS One. 2008;3(7):1–7. 10.1371/journal.pone.0002680 PMC2481297 18648549

