# Supplementary material for: Coral cell separation and isolation by fluorescence-activated cell sorting (FACS)
Source: BMC Cell Biol. 2017 Aug 29;18:30. doi: 10.1186/s12860-017-0146-8 (PMC5575905; doi:10.1186/s12860-017-0146-8)
Supplement: Additional file 1: Figure S1. — Comparison of coral cell dissociation. P. damicornis cell suspension was collected either mechanically using a fine blade as described in the methods (A) or also incubated at room temperature with Trypsin 0.025% of Trypsin-EDTA in 3.3XPBS (B). Cell suspension was labeled with DAPI, CellRox, and LysoTracker Deep Red, and analyzed using SH800S Cell Sorter. Among the cells isolated without Trypsin (A) about 8% were positive to DAPI whereas 31% were positive to DAPI isolated with Trypsin (B), in the rectangular gate. This DAPI stain results corroborates our observation with Trypan Blue by light microscopy (data not shown). Figure S2. Symbiodinium positive population validation. In addition to the sorted cells microscopy, to validate that the population observed on the far red and green channels without labeling is Symbiodinium we used wild type (WT) Aiptasia that contains Symbiodinium and stable strains that stably grow for more than a decade without any symbiotic algae (APO). It can be observed that the gate of Symbiodinium positive cells is almost empty of cells (Right panel; 0.17%) in the APO strain while there are about 15% of cells in the gate of the wild type strain. Figure S3. Green fluorescent beads control for phagocytosis assays. To validate that the green fluorescent beads positive cells are the gated populations (Figs. 2, 5, 6) we ran the beads alone. Due to the size of the beads (1 μm) most of them that are not conjugated to cells are gated out on the size and granularity gate (FSC, SSC). Only 8 out of 10,000 events are positive in the gate. (DOCX 577 kb) [file 12860_2017_146_MOESM1_ESM.docx]

**Additional file 1 for the manuscript:**

**Title: Coral cell separation and isolation by fluorescence-activated cell sorting (FACS)**

**Figure S1.**


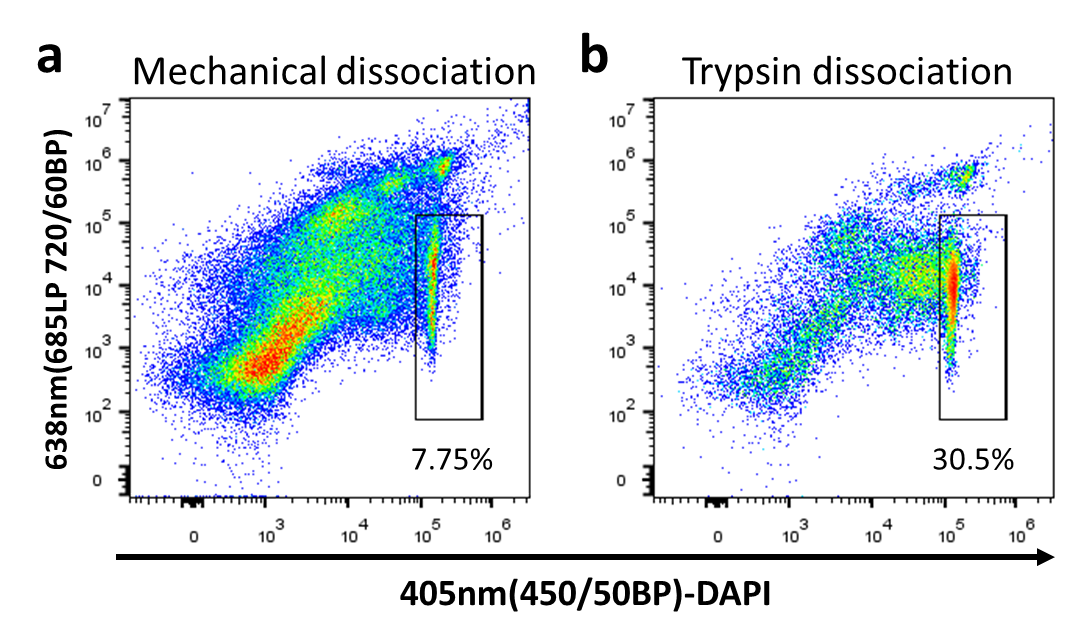


**Figure S1. Comparison of coral cell dissociation.** *P. damicornis* cell suspension was collected either mechanically using a fine blade as described in the methods (A) or also incubated at room temperature with Trypsin 0.025% of Trypsin-EDTA in 3.3XPBS (B). Cell suspension was labeled with DAPI, CellRox, and LysoTracker Deep Red, and analyzed using SH800S Cell Sorter. Among the cells isolated without Trypsin (A) about 8% were positive to DAPI whereas 31% were positive to DAPI isolated with Trypsin (B), in the rectangular gate. This DAPI stain results corroborates our observation with Trypan Blue by light microscopy (data not shown).

**Figure S2.**


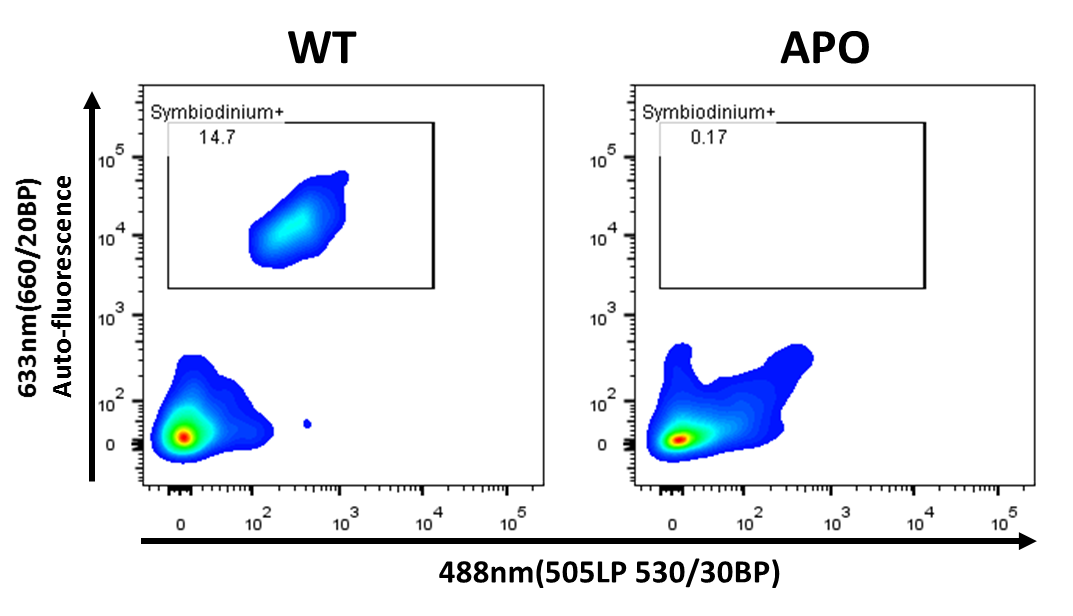


**Figure S2. *Symbiodinium* positive population validation.** In addition to the sorted cells microscopy, to validate that the population observed on the far red and green channels without labeling is *Symbiodinium* we used wild type (WT) *Aiptasia* that contains *Symbiodinium* and stable strains that stably grow for more than a decade without any symbiotic algae (APO). It can be observed that the gate of *Symbiodinium* positive cells is almost empty of cells (Right panel; 0.17%) in the APO strain while there are about 15% of cells in the gate of the wild type strain.

**Figure S3.**


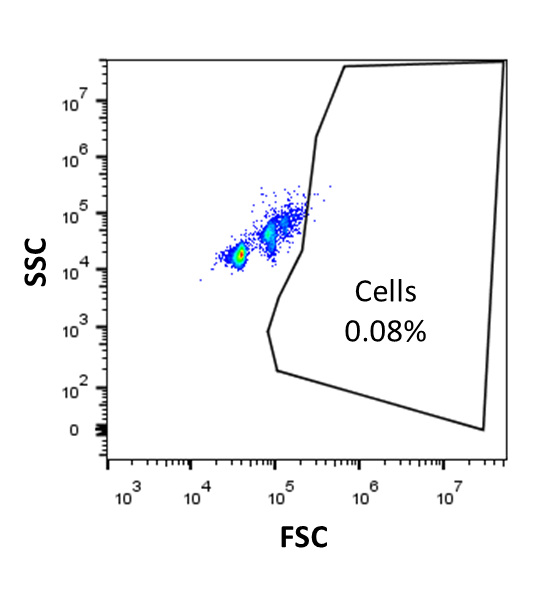


**Figure S3. Green fluorescent beads control for phagocytosis assays.** To validate that the green fluorescent beads positive cells are the gated populations (Figures 2, 5, 6) we ran the beads alone. Due to the size of the beads (1 µm) most of them that are not conjugated to cells are gated out on the size and granularity gate (FSC, SSC). Only 8 out of 10,000 events are positive in the gate.
